# Global metabolomics profiling of glucuronides in human plasma, fecal, and cerebrospinal fluid samples

**DOI:** 10.1007/s00216-025-06082-w

**Published:** 2025-09-01

**Authors:** Ioanna Tsiara, Mario S. P. Correia, Fan Yang, Weiming Zeng, Pauline Seeburger, Belén Hervás Povo, Iben Lundgaard, Manuel Menéndez-González, Miroslav Vujasinovic, J.-Matthias Löhr, Daniel Globisch

**Affiliations:** 1 Department of Chemistry - BMC, Science for Life Laboratory, Uppsala University, Box 576, 75124 Uppsala, Sweden; 2 Department of Experimental Medical Science, Lund University, 22362 Lund, Sweden; 3 Wallenberg Centre for Molecular Medicine, Lund University, 22362 Lund, Sweden; 4 Department of Medicine, Universidad de Oviedo, 33006 Oviedo, Asturias Spain; 5 Instituto de Investigación Sanitaria del Principado de Asturias, 33011 Oviedo, Spain; 6 Department of Medicine Huddinge, Karolinska Institute, Stockholm, Sweden; 7 Department for Upper Abdominal Diseases, Karolinska University Hospital, Stockholm, Sweden; 8 Department of Clinical Science, Intervention and Technology (CLINTEC), Karolinska Institute, Stockholm, Sweden

**Keywords:** Glucuronides, Phase II modifications, Enzymatic synthesis, Metabolomics, Gut microbiota

## Abstract

Glucuronidation is the major phase II biotransformation reaction that facilitates the clearance of exogenous compounds from the human body. Glucuronidated metabolites have been investigated in urine samples at a broad scale; however, their characterization in other human biospecimens is underexplored. Our study has now performed a comprehensive profiling of glucuronides in plasma, fecal, and cerebrospinal fluid (CSF) of humans. We performed a mass spectrometry-based analysis that combines enzymatic hydrolysis with a *β*-glucuronidase to selectively cleave the glucuronic acid moiety, a developed in-house glucuronide identification pipeline, and enzymatic synthesis of standard metabolites. In total, we identified 32 glucuronidated metabolites across the three sample types in both negative and positive mass spectrometry ionization modes using advanced MS/MS fragmentation analysis. We have utilized a straightforward enzymatic synthesis of glucuronidated metabolites for annotation at the highest confidence level. Among the identified conjugates, we detected glucuronides of different compound classes including drugs, bile acid derivatives, steroid conjugates, and phenolic compounds. Unexpectedly, we validated the glucuronides of acetaminophen and propofol in CSF samples, the latter representing a novel observation that highlights the importance of investigating phase II metabolites in uncommon sample types.

**Graphical Abstract:**

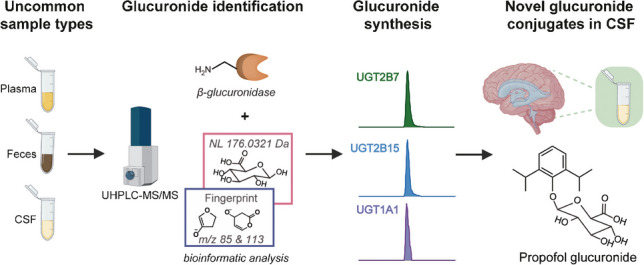

**Supplementary Information:**

The online version contains supplementary material available at 10.1007/s00216-025-06082-w.

## Introduction

Xenobiotics include dietary compounds that are metabolized at different sites in the human body. The primary metabolic processes include phase I and phase II conversion, which promote excretion by increasing the hydrophilicity of compounds. The most common phase II biotransformation reaction is glucuronidation, catalyzed by uridine diphosphate (UDP)-glucuronosyltransferases, which accounts for 40–55% of xenobiotic metabolism [1]. Glucuronidated metabolites are derived from various sources like diet, drugs, and microbial metabolism [2–5]. Recent studies highlight the gut microbiota’s crucial role in xenobiotic metabolism, influencing both drug clearance and disease physiology [6, 7]. Despite the importance of glucuronidation as a metabolic process, comprehensive untargeted investigation of glucuronidated metabolites in human samples beyond urine remains scarce. While plasma reflects the systemic circulation of metabolites, global analysis of plasma glucuronides is limited. So far, most research of glucuronidated compounds focuses on the targeted analysis of specific drug glucuronide conjugates or targeted bioactive molecules of pharmacological and nutritional interest [8–14]. Similarly, the intestine hosts the largest population of bacteria; thus, the study of fecal samples is essential to understand the host-microbiota co-metabolism. The sample type also lacks an in-depth analysis of fecal glucuronides. It is important to note that the gut microbiota exhibits *β*-glucuronidase activity that hydrolyzes glucuronidated metabolites and releases the aglycon, before excretion in the fecal matter [15–17].

In our previous studies, we have extensively investigated the glucuronides excreted in human urine samples utilizing *β*-glucuronidase sample pre-treatment and bioinformatic tools for their selective identification [2, 18, 19]. Herein, we expand our mass spectrometric investigation to include glucuronidated metabolites in plasma, feces, and cerebrospinal fluid (CSF). Additionally, we have expanded our analysis to the positive mass spectrometry ionization mode to increase the discovery coverage of this compound class. We have developed an optimized script in R for the selective identification of glucuronidated metabolites, facilitating a more comprehensive characterization of this metabolite class. Lastly, to optimize metabolite identification, we have performed a straightforward enzymatic synthesis of glucuronidated metabolites that were used for co-injection experiments.

## Materials and methods

Information for the sample collection can be found in “Ethics approval and source of biological material”.

### General

All reagents and solvents were purchased from Sigma-Aldrich or Fischer Scientific and were used without further purification. HPLC grade solvents were used for HPLC purification and mass spectrometry grade for UHPLC-ESI–MS analysis. All biochemical reactions were performed with HPLC or LC–MS grade solvents. Solutions were concentrated in vacuo on a Speedvac Concentrator Plus System (Eppendorf, Hamburg, Germany). High-resolution mass spectra were acquired on a Maxis II ETD Q-TOF mass spectrometer (Bruker Daltonics, Germany) using an electrospray ionization (ESI) source with an Elute UHPLC (Bruker Daltonics, Germany) system and equipped with a Waters ACQUITY UPLC HSS T3 column (1.8 μm, 100 × 2.1 mm).

### Sample preparation

#### Sample group I

##### CSF samples

Exactly 50 µL of CSF (n = 21) samples was aliquoted into 1.5 mL Eppendorf tubes. To each sample, 10 µL of internal standard mixture (5 μg/mL of tyrosine-^13^C_9_,^15^N, 10 μg/mL of phenylalanine-^13^C_9_,^15^N, and 20 μg/mL of valine-^13^C_5_) and 240 µL of ice-cold methanol were added. The samples were vortexed and stored at − 20 °C for 1 h to precipitate proteins. Next, the samples were centrifuged (14,500 rpm, 5 min, 4 °C), and the supernatants were collected and dried under vacuum in a Speedvac for 1.5 h. The dried extracts were stored at − 20 °C until enzymatic assays were performed.

#### Sample group II

##### Plasma samples

Plasma samples (n = 21) were prepared using the same protocol as described for CSF samples.

##### Fecal samples

Fecal samples (n = 19) were processed under sterile conditions in a UV-sterilized hood, on dry ice. All surfaces and tools were disinfected with 70% ethanol, and dry ice was used throughout the sample preparation to prevent sample thawing. Approximately 50 mg of each frozen fecal sample was transferred to pre-weighed 2 mL Eppendorf tubes, and the fresh weight (FW) was recorded. Next, the samples were freeze-dried (Labconco, FreeZone 4.5 L Benchtop Freeze-Dryer) overnight. The dry weights (DW) of the samples were determined, and the extraction volumes were adjusted accordingly. Each sample was reconstituted in dimethyl sulfoxide (DMSO), using a volume based on the dry weight (µL DMSO = DW/1.2), followed by the addition of Milli-Q water (µL Milli-Q = DMSO volume × 19), yielding a final concentration of 60 mg DW/mL. The samples were sonicated for 5 min and centrifuged (14,500 rpm, 5 min, 4 °C). Supernatants were collected and aliquoted into Eppendorf tubes, then concentrated under vacuum in a Speedvac and stored at − 20 °C. The dried extracts were reconstituted in 50 µL Milli-Q water, followed by the addition of 240 µL ice-cold methanol and 10 µL internal standard mixture (5 μg/mL of tyrosine-^13^C_9_,^15^N, 10 μg/mL of phenylalanine-^13^C_9_,^15^N, and 20 μg/mL of valine-^13^C_5_). Samples were vortexed and stored at − 20 °C for 1 h to precipitate proteins. Then the samples were centrifuged (14,500 rpm, 5 min, 4 °C), and the supernatants were collected and dried under vacuum. The dried extracted samples were stored at − 20 °C until enzymatic assays were performed.

### Enzymatic assay

For the enzymatic assay, the plasma, fecal, and CSF dried extracts were redissolved in 75 mM phosphate buffer (100 µL for plasma and CSF samples and 200 µL for fecal samples). Following reconstitution, pooled samples were prepared by combining equal volumes of plasma, fecal, and CSF extracts for each condition: enzymatic assay (EA) and control group. To start the enzymatic reaction, 25 µL of 100 U B-One enzyme (Kura Biotech, B-One-10 mL, lot no. 1051) was added to the enzymatic assay groups, and 25 µL of denatured enzyme (Thermomixer, 99 °C, 1500 rpm, 30 min) was added to the control groups. The enzymatic assay and control groups for each sample type were incubated for 16 h (Thermomixer, 37 °C, 300 rpm).

Aliquots of 25 µL were collected at timepoints 0 h for the control group and 16 h for the enzymatic assay group and immediately quenched with 100 µL of ice-cold methanol. The samples were kept on ice for 15 min and then centrifuged (13,400 rpm, 5 min, 4 °C). Supernatants were collected and concentrated using a vacuum concentrator (SpeedVac) for 1.5 h. The pellets were reconstituted in 50 µL of 5% acetonitrile in Milli-Q water (v/v, 5:95), vortexed vigorously for 20 s, and centrifuged again (13,400 rpm, 5 min). The final supernatants were transferred into HPLC vials and analyzed using UHPLC-MS/MS. All control and EA groups per sample were injected.

### Enzymatic synthesis of glucuronides

Six compounds (five phenolic and one bile acid) were treated with UGT1A1, UGT2B7, and UGT2B15 (Electronic Supplementary Material Fig. S1). The strategy to test three enzymes is based on maximizing the substrate promiscuity to successfully synthesize the reference compounds. The enzymatic synthesis consisted of a mixture of 4 µL of UGT reaction mixture solution A (Corning), 10 µL of Corning Gentest UGT reaction mix solution B (Corning), 10 µL of the desired compound to synthesize, at a concentration of 250 µM, and 10 µL of either Human UGT1A1 supersomes, Human UGT2B7 supersomes, Human UGT2B15 supersomes (all from Corning), or water (as negative control). The reaction mixture was incubated at 37 °C overnight (16 h). Methanol (100 µL) was added to stop the reaction and to precipitate the enzymes. The mixture was kept at − 20 °C for 20 min. After centrifugation at 13,400 rpm for 5 min, the supernatant was transferred to an LC–MS vial for analysis.

### UHPLC-MS analysis

The UHPLC–MS/MS analysis was conducted using a maXis II ETD Q-TOF mass spectrometer (Bruker Daltonics, Germany) coupled with an Elute UHPLC system (Bruker Daltonics, Germany) and equipped with an electrospray ionization (ESI) source. Chromatographic separation was carried out on an ACQUITY UPLC HSS T3 column (1.8 μm, 100 × 2.1 mm) from Waters Corporation. The mobile phase consisted of two solvents: solvent A was Milli-Q water with 0.1% formic acid and solvent B was LC–MS-grade methanol containing 0.1% formic acid. The column temperature was maintained at 40 °C, and the autosampler was kept at 4 °C. A flow rate of 0.20 mL/min was used, with an injection volume of 5 μL. The gradient elution program was as follows: 0–2 min, 0% B; 2–15 min, 0–100% B; 15–16 min, 100% B; 16–17 min, 100–0% B; and 17–21 min, 0% B. The system was operated through the Compass HyStar software package (Bruker Daltonics, Germany). The MS acquisition was performed in negative and positive ionization mode, with a mass detection range of *m/z* 50–1200. Data acquisition was carried out in AutoMS/MS mode (data-dependent acquisition, DDA) with a 0.5 s cycle time and a ramped collision energy varying from 20 to 50 eV. For the MRM experiments, the collision energy was set at 10, 20, or 40 eV. Internal calibration was performed at the start of each LC–MS run using a sodium formate solution (10 mM in a 1:1 mixture of 2-propanol and water) within the 0.10–0.31 min time segment. Each plasma, fecal, and CSF sample (control and EA group) was injected four times into the UHPLC-MS/MS with a randomized sample analysis list.

### Data analysis

First, the raw data were converted to mzML using MS convert (ProteoWizard). The two groups, control and EA, were processed in R using Spectra and MetaboAnnotation packages [20]. The codes are accessible in GitHub (https://github.com/Globischlab/Glucuronides-MSmatch). The data processing workflow consisted of the following steps:

i. Search for aglycon: the precursor ions from the control samples were extracted to calculate the theoretical mass of the aglycons, with the loss of the glucuronide moiety (176.0321 Da, C_6_H_8_O_6_). The theoretical masses of the aglycons were matched with the precursor ions obtained from the EA samples, where the glucuronide moiety is cleaved during the enzymatic assay.
ii. Aglycon and glucuronide identification by precursor mass: From *step i*, each precursor ion from the EA samples was theoretically paired with one precursor ion from the control samples. The paired precursor ions from the control samples were screened against HMDB and PubChem glucuronides subtracted dataset by precursor masses, which assigns putative annotations. Similarly, the paired precursor ions from the EA samples were screened against HMDB with ion masses for the aglycon annotation.
iii. MS/MS matching: The glucuronide moiety presents the characteristic fragmentation in the negative ionization mode [18]. Therefore, we searched for the fragments *m/z* 85.0295, 113.0244 in the spectra, then separately analyzed the precursor and fragmentation information. In the positive ionization mode, no specific fingerprint ions were observed for the glucuronidated metabolites.

## Results and discussion

To determine the presence and identity of glucuronidated metabolites at a global level in the three uncommon sample types of plasma, feces, and CSF, we have applied our enzymatic assay to metabolite extracts from these sample types. Towards selectively identifying the metabolite structure, we have improved our sample treatment, developed a script for the bioinformatics analysis, and introduced an enzymatic synthesis of standard metabolites.

### Identification of glucuronide conjugates in plasma, feces, and CSF samples

**Fig. 1:**
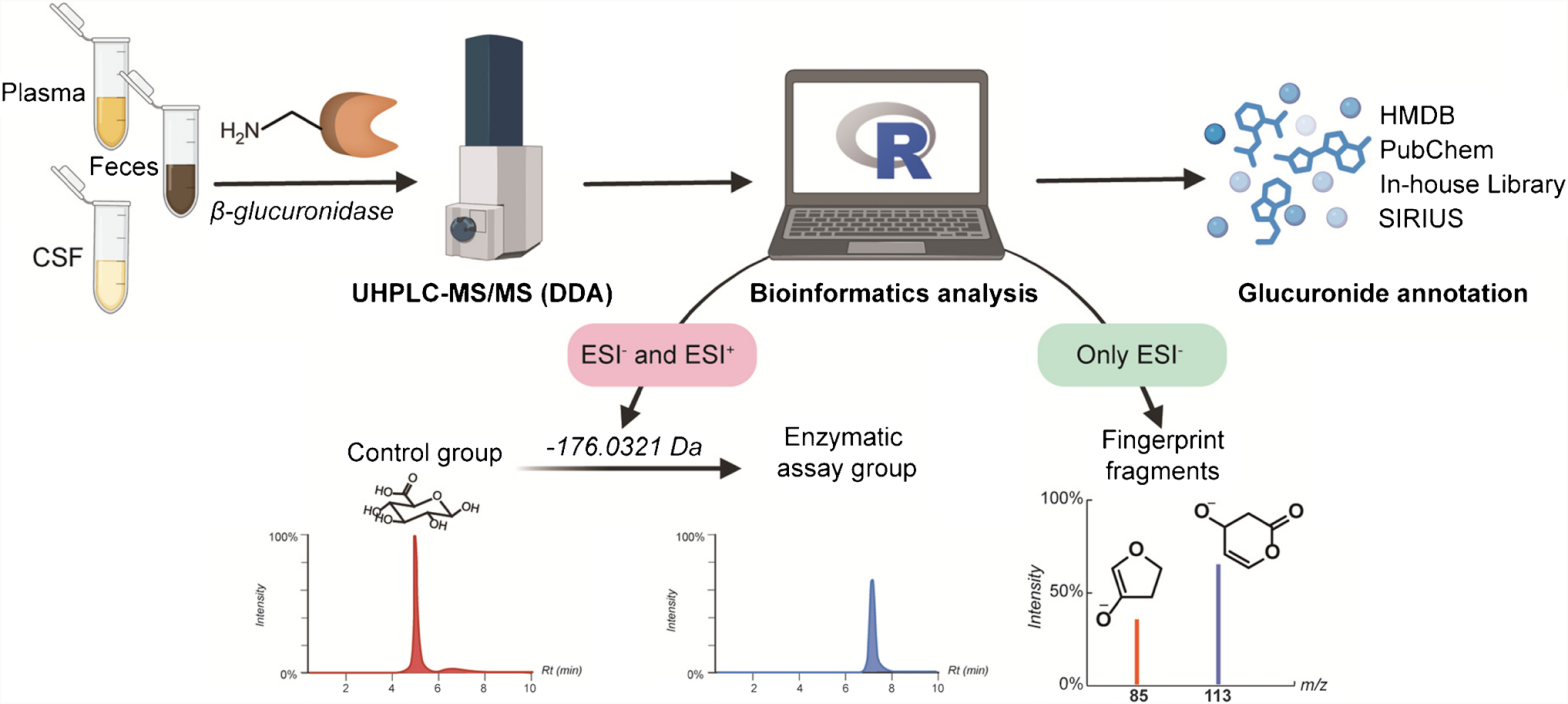
Our developed workflow for the identification of glucuronides in plasma, fecal, and CSF samples. The samples are treated with the B-One *β*-glucuronidase for the selective identification of glucuronidated compounds. The UHPLC-MS/MS acquisition is performed in data-dependent mode (DDA), using a ramped collision energy of 20–50 eV, in both positive (ESI^+^) and negative (ESI^−^) ionization modes. Through optimized bioinformatic analysis in R, we identify the features where the glucuronic acid loss occurs (176.0321 Da) between the control and the enzymatically treated samples. Additionally, for ESI^−^ we screen for the characteristic most abundant fingerprint fragments of glucuronic acid in this collision energy (*m/z* 85.0295, 113.0244). After combining these glucuronide discovery pipelines, we perform library matching using HMDB, PubChem, SIRIUS, and our in-house library for the glucuronide or the aglycon

**Fig. 2:**
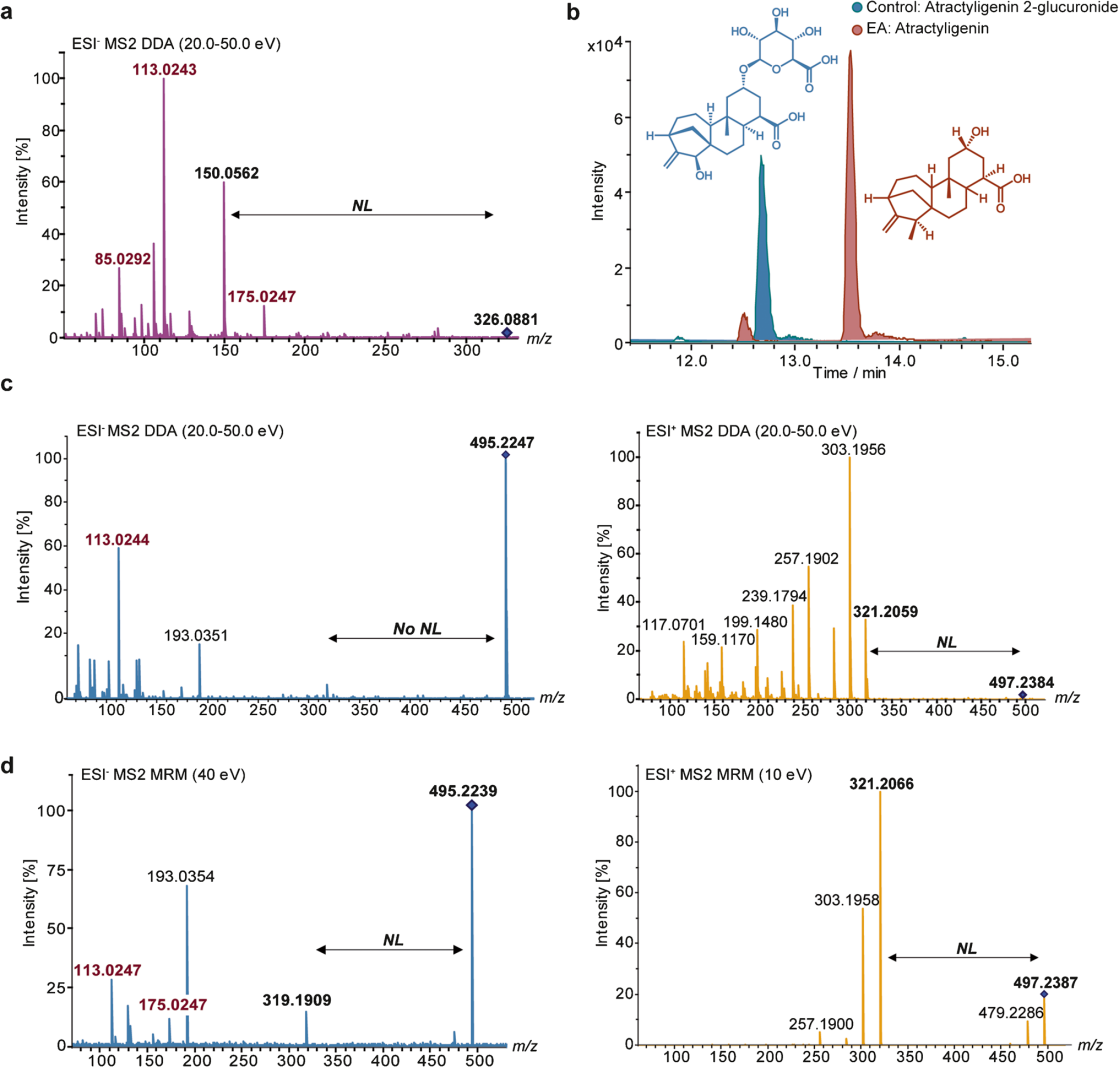
Example identification of acetaminophen glucuronide (exact mass = 327.0955 Da) and atractyligenin 2-glucuronide (exact mass = 496.2308 Da). **a** MS/MS spectra of acetaminophen glucuronide (*m/z* 326.0881) in ESI^−^ using DDA at 20–50 eV; **b** Extracted ion chromatograms (EICs) of atractyligenin 2-glucuronide (*m/z* 495.2238) in the control group and its aglycon, atractyligenin (*m/z* 319.1920) in the enzymatic assay group in ESI^−^; **c** MS/MS spectra of atractyligenin 2-glucuronide in ESI^−^ (left) and ESI^+^ (right) using DDA at 20–50 eV; **d** MS/MS spectra of atractyligenin 2-glucuronide in MRM mode using 40 eV in ESI^−^ (left) and 10 eV in ESI^+^ (right). NL represents the neutral loss of 176.0321 Da. The characteristic fingerprint fragments of the glucuronic acid are highlighted in red color

In total, 32 glucuronide conjugates were identified across all three sample types using a combination of accurate mass detection in positive (ESI^+^) and negative (ESI^−^) mass spectrometry ionization modes, enzymatic hydrolysis with a recombinant *β*-glucuronidase, fragmentation analysis, and database matching with either HMDB, PubChem, SIRIUS, or our in-house library (Fig. 1) [21–23]. We have performed enzymatic assays (EA) separately for the three biospecimens with the recombinant *β*-glucuronidase B-One to selectively convert glucuronidated metabolites for specific analysis. Features in the control group with a mass difference of Δ*m/z* 176.0321 Da compared to their corresponding aglycons in the EA group were considered potential glucuronides. In ESI^−^, we further screened for the characteristic fingerprint ions that are most dominant (*m/z* 85.0295, 113.0244) in data-dependent acquisition (DDA) using a ramped collision energy (CE) of 20–50 eV as previously optimized for this compound class [2, 19, 24]. Although the fragment ion at *m/z* 175.0248 served as a diagnostic marker in our previous analysis of urine samples, in the current dataset it was not consistently observed, probably due to the low abundance of the glucuronidated molecules in these biospecimens [2]. Similarly, the expected neutral loss (NL) of 176.0321 Da was often absent in the MS/MS spectra of the putative glucuronides, reflecting either low precursor ion intensity or matrix effects. An ideal example is acetaminophen glucuronide that contained all three fingerprint fragments (*m/z* 85.0295, 113.0244, and 175.0248) and the characteristic neutral loss, showcasing the level of spectral evidence desirable for high confidence annotation of glucuronides (Fig. 2a). However, these complete fragmentation patterns were the exception in our dataset since most detected glucuronides lacked one or more of these features, complicating confident structural confirmation. In positive ionization mode, no distinct glucuronide-specific fragment ions were observed, limiting the use of MS/MS for structural confirmation of metabolites in this mode.

A representative example of the complexity of the identification in our datasets is atractyligenin 2-glucuronide, which was detected in the plasma samples in both ESI^−^ and ESI^+^. The specificity of the *β*-glucuronidase treatment provides strong evidence for glucuronide conjugation, with a 100% decrease of atractyligenin 2-glucuronide in the EA treated sample demonstrating complete hydrolysis (Fig. 2b). This glucuronidated molecule displayed no characteristic NL of 176.0321 Da in DDA with CE 20–50 eV in ESI^−^ (Fig. 2c, left). However, for this compound we observed the fingerprint fragment of *m/z* 113.0244 (intensity of 60%). While the characteristic NL of 176.0321 Da was not observed in the MS/MS spectra, likely due to low precursor ion intensity in this complex biological matrix, the combined evidence supports annotation as atractyligenin 2-glucuronide (confidence level (CL) 2). The same glucuronide was examined in ESI^+^, where the calculated decrease corresponded to 92% post-treatment (Electronic Supplementary Material Fig. S2). This difference can be explained due to the ionization properties of the glucuronides in the respective mode. The MS/MS spectra in ESI^+^ mode also demonstrated the distinct NL and the presence of the aglycon fragment (*m/z* 321.2066), confirming the identification (Fig. 2c, right).

Then, we sought to evaluate different CEs for the conjugated atractyligenin, focusing on lower CEs of 10 eV and 20 eV, that have been reported to yield informative fragmentation for sugar moieties, particularly in certain glycosides [25]. However, for ESI^−^, no fragmentation occurred at these CEs, highlighting the need for higher CEs or a ramped approach as utilized by us to account for the structural diversity of glucuronidated metabolites (Electronic Supplementary Material Fig. S3) [24, 26]. When we applied a higher CE of 40 eV, the NL was observed, with a fragment ion at *m/z* 319.1929 (intensity 15%; Fig. 2d, left). Additionally, the characteristic fragment at *m/z* 175.0247 was detected with a relative intensity of 12%. For ESI^+^, the lower CE of 10 eV exhibited a clear neutral loss fragmentation ion that corresponds to the aglycon (*m/z* 321.2066), which is the highest intensity ion (100%; Fig. 2d, right). While a simple neutral loss analysis seems straightforward, this example demonstrates how challenging the investigation of the compound class of glucuronides is at a global level and in uncommon sample types.

### Enzymatic synthesis of authentic standards

**Fig. 3:**
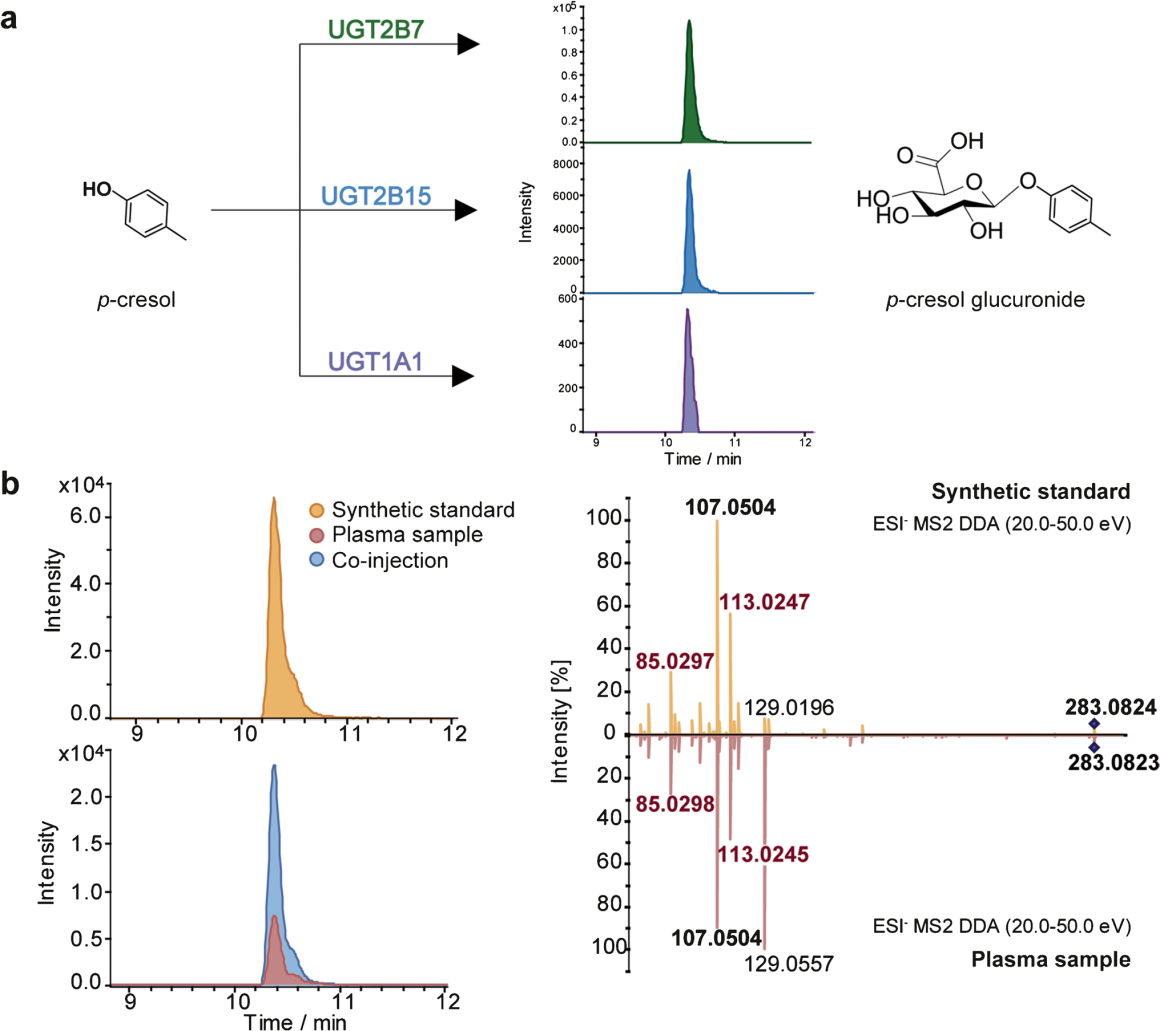
Enzymatic synthesis of *p*-cresol glucuronide (*m/z* 283.0822) from the authentic standard of *p*-cresol (*m/z* 107.0501) for UHPLC-MS/MS co-injection experiment in ESI^−^. **a** *p*-cresol glucuronide synthesis was performed using three UGT isoenzymes: UGT2B7, UGT2B15, and UGT1A1; **b** Co-injection experiment of the synthesized *p*-cresol glucuronide in a plasma sample (left) and MS/MS spectra comparison of the synthetic standard and the plasma sample (right). The characteristic fingerprint fragments of the glucuronic acid are highlighted in red color

A major bottleneck in the investigation of glucuronides is the lack of commercially available standards, which limits the confidence in compound identification. The synthesis of glucuronides both chemically and enzymatically is a well-established concept; however, the availability of reference molecules remains limited [27–29]. By using three recombinant UGT isoenzymes (UGT1A1, UGT2B7, UGT2B15), we have performed a straightforward enzymatic synthesis protocol for five phenolic compounds (phenol, *p*-cresol, 4-methoxyphenol, 4-acetamidophenol, ferulic acid) and one bile acid (cholic acid). This assay led to the successful glucuronidation of five of these selected substrates (Electronic Supplementary Material Table S1). These metabolites were selected based on prior detection in biological matrices or their relevance in microbiota-host interactions. The enzymatic synthesis of standards of these compounds represents a proof-of-concept strategy to obtain reference compounds in general. As an example for structure validation, we present the synthesis of *p*-cresol glucuronide, a known microbiota-derived metabolite that we detected in plasma and in low abundance in fecal samples (Fig. 3a). The results demonstrated that *p*-cresol glucuronide formation was primarily catalyzed by UGT2B7, with moderate and minimal conversion observed for UGT2B15 and UGT1A1, respectively. Next, we performed a co-injection experiment of the synthetic *p*-cresol glucuronide with a pooled plasma sample. The retention time and fragmentation spectra were perfectly matched (Fig. 3b). The chemical structure of this key microbiota-derived metabolite is thus validated at the CL1, providing high confidence annotation in the absence of commercial reference compounds.

### Exploring glucuronidation across sample types

**Fig. 4:**
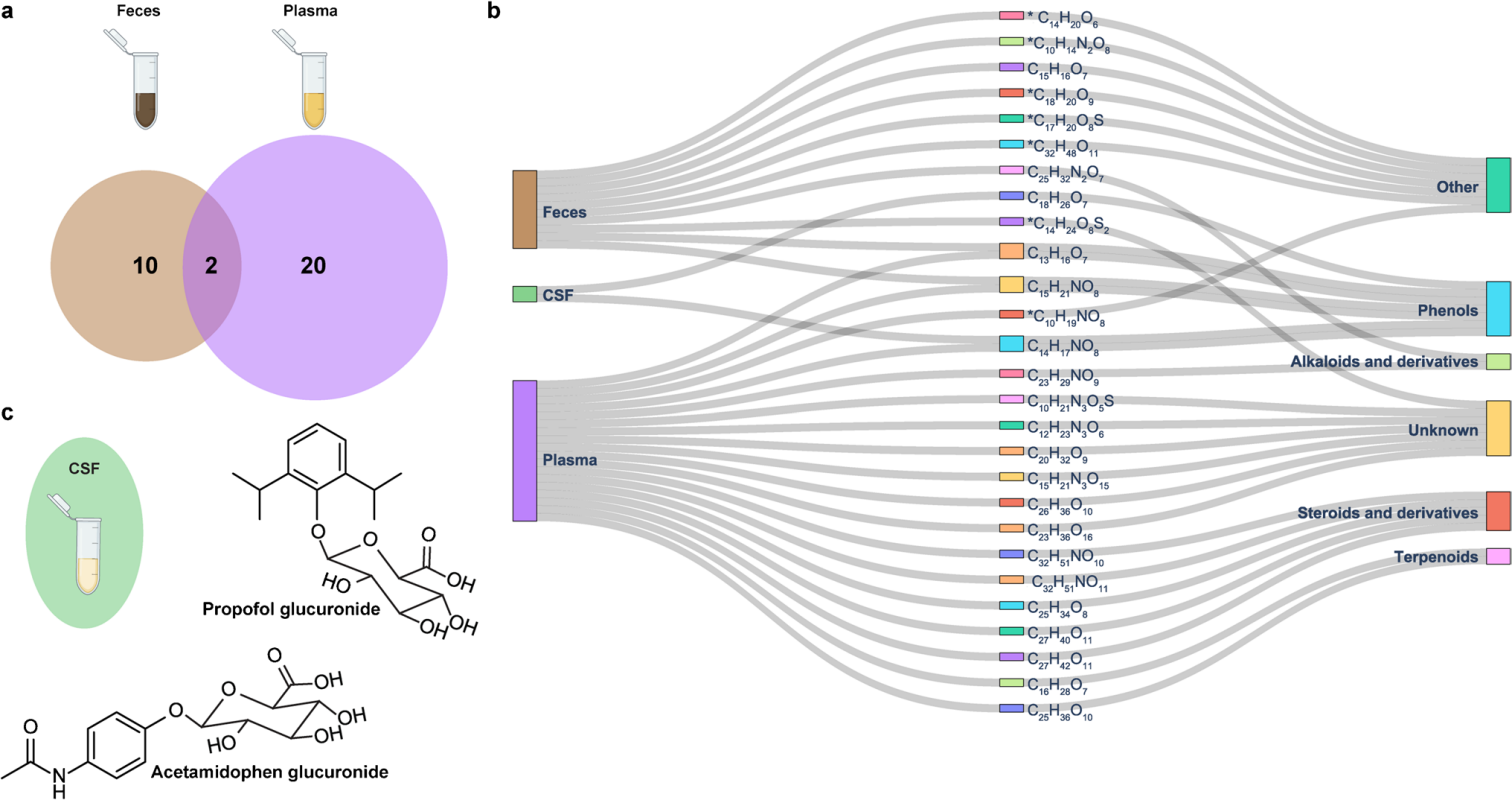
Overview of glucuronidated metabolites overlap and distribution across plasma, fecal, and CSF samples. **a** Venn diagram with the identified glucuronides in plasma and fecal samples derived from the same individuals with high risk for pancreatic cancer (sample group II); **b** Sankey diagram illustrating the distribution and classification of identified glucuronides across the three biospecimens: plasma, CSF, and feces. The nodes on the left represent the biospecimen sources, which are connected to the chemical formulas of detected glucuronides (center nodes) and further grouped into broader chemical classes (nodes on the right) based on structural annotation of the aglycon. Each link represents a unique metabolite mapping between the categories, with line thickness reflecting detection frequency. The chemical formulas with an asterisk (*) represent a calculated chemical formula from the aglycon (aglycon + C_6_H_8_O_6_); **c** Structures of propofol glucuronide (exact mass = 354.1684 Da) and acetaminophen glucuronide (exact mass = 327.0955 Da) detected in CSF samples of postmortem cases (sample group I)

We identified 10 glucuronides in fecal, 20 in plasma, and 2 in CSF samples with the highest confidence of a glucuronide present (Fig. 4a, Electronic Supplementary Material Table S2). The annotation confidence varied, with one metabolite validated at the highest confidence level (CL 1), three assigned at CL 2, while the other metabolites remained putative (CL 3). A range of chemical classes was detected in the three sample types, including drugs, bile acid derivatives, steroid conjugates, and phenolic compounds (Electronic Supplementary Material Table S3). As expected, our results show a higher number of glucuronidated metabolites in plasma compared to feces, which aligns with the metabolite data available in the Human Metabolome Database (HMDB) [21].

Initially, we sought to explore the glucuronidation profiles particularly of the plasma and fecal samples collected from high-risk individuals for pancreatic cancer (sample group II) [30, 31]. In plasma, we identified several glucuronidated metabolites with high confidence, including acetaminophen glucuronide that was detected in all three sample types. Bile acid glucuronides like glycolithocholic acid and glycochenodeoxycholic acid were exclusively detected in plasma (Fig. 4b). Bile acid metabolism has been implicated in pancreatic cancer progression, and altered bile acid profiles have been linked to microbial imbalances in cancer patients [32]. However, specific studies on bile acid glucuronides linked to disease development are limited. Additionally, several steroid metabolites including 6-dehydrotestosterone glucuronide, tetrahydroaldosterone-3-glucuronide, and cortolone-3-glucuronide were detected in plasma. Similarly to the bile acid conjugates, there are no studies focusing on steroid glucuronides in a pancreatic disease context; however, previous studies have shown that steroid hormones are dysregulated in male patients with this cancer diagnosis [33, 34].

In fecal samples, we identified important phenolic compounds, like *p*-cresol glucuronide and phenol-substituted compounds with chemical formulas C_15_H_16_O_7_ and C_15_H_21_NO_8_. Additionally, a prostaglandin analog (C_32_H_48_O_11_) and sulfur-containing metabolites, putatively annotated as C_17_H_20_O_8_S and C_14_H_24_O_8_S_2_, were detected. *p*-Cresol glucuronide is a metabolite also detected in the plasma samples, suggesting potential systemic circulation of gut-derived microbial metabolites [35]. This aligns with previous studies indicating that gut microbiota-derived metabolites can contribute to inflammation and pancreatic cancer development [36].

Interestingly, we detected the two drug metabolite conjugates acetaminophen glucuronide and propofol glucuronide (CL 2) in the CSF of postmortem cases with Alzheimer’s disease (sample group I; Fig. 4c, Electronic Supplementary Material Fig. S4). The identification of propofol glucuronide in CSF samples represents a novel and unexpected finding. To the best of our knowledge, this is the first report of any glucuronidated drug metabolite detected in human CSF. Previous pharmacokinetic studies have reported the presence of unconjugated propofol in CSF during anesthesia. However, glucuronide conjugates have thus far only been quantified in blood or urine [37–39]. The detection in postmortem CSF may reflect not only systemic exposure but also altered blood–CSF barrier integrity or impaired clearance mechanisms in neurodegenerative diseases such as Alzheimer’s disease. Since we investigated a pooled CSF sample, we identified the individual sample of the postmortem subjects with Alzheimer’s disease in separate UHPLC-MS analyses of each patient. We detected propofol glucuronide in only one individual, and this subject was confirmed to have undergone general anesthesia before their death. These results underscore the sensitivity of our method and highlight the potential of innovative, untargeted metabolomics for detecting phase II metabolites in CNS-derived fluids. Furthermore, this observation may hold value in forensic contexts, where detection of conjugated drug metabolites in CSF could assist in reconstructing recent pharmacological interventions.

Noteworthy, the untargeted identification of glucuronides in these biospecimens proved challenging due to the low abundance of this phase II metabolite class in these complex matrices. As mentioned previously, the intensity of the characteristic fragments (*m/z* 85.0295, 113.0244) in ESI^−^ was low, and the NL in MS/MS was absent in most of the cases for both ionization modes. The ramping CE of 20–50 eV ensures detection of both low-energy fragments, characteristic of labile glucuronide bonds (including potential neutral losses of 176.0321 Da), and higher-energy fragments that facilitate structural elucidation of the corresponding aglycon.

An important limitation of investigating glucuronides in fecal samples is that the weak alkaline environment in the bile and the small intestine can promote the hydrolysis of alkali-labile acyl glucuronidated metabolites [40]. Furthermore, bacterial *β*-glucuronidases and carboxylesterases can cleave the acyl *β*-glucuronidated metabolites, further reducing their stability [41]. These biochemical reactions result in the rapid reabsorption of the aglycon and promote enterohepatic circulation. Thus, certain glucuronidated metabolites may not be excreted through feces and therefore remain undetected in fecal samples. Similarly, the analysis of glucuronides in CSF is also challenging due to the low abundance of this metabolite class. The main reason is possibly resulting from their restricted transport across the blood–CSF barrier [42]. Additionally, certain glucuronides may form within the central nervous system, but their polar properties generally limit their diffusion into the CSF [43].

## Conclusion

In summary, in this study we performed a mass spectrometric global profiling of glucuronidated metabolites in plasma, fecal, and CSF samples. We developed a combined approach utilizing enzymatic sample pre-treatment with a *β*-glucuronidase to selectively investigate this compound class, bioinformatic analysis, and enzymatic synthesis of glucuronide standards. While glucuronidation was predominantly detected in plasma samples compared to the other two sample types, the metabolite abundance was low. In total, we have successfully detected 32 glucuronide conjugates in all three sample types and synthesized five reference standards as proof of concept for annotation of metabolites at the highest level of confidence. Interestingly, we identified two drug conjugates present in the CSF samples, acetaminophen and propofol glucuronide, which have never been detected before that demonstrates the sensitivity and novelty of our analytical approach in detecting glucuronidated metabolites in uncommon sample types.

## Supplementary Information

Below is the link to the electronic supplementary material.

Supplementary file1 (PDF 649 KB)

## Data Availability

The manuscript has data included as electronic supplementary material. The raw data are available upon request.
