# Supplementary material for: Global metabolomics profiling of glucuronides in human plasma, fecal, and cerebrospinal fluid samples
Source: Anal Bioanal Chem. 2025 Sep 1;418(16):5059–70. doi: 10.1007/s00216-025-06082-w (PMC13423926; doi:10.1007/s00216-025-06082-w)
Supplement: Supplementary file 1 — Supplementary file1 (PDF 649 KB) [file 216_2025_6082_MOESM1_ESM.pdf]

Supporting Information

# Global Metabolomics Profiling of Glucuronides in Human Plasma, Fecal, and Cerebrospinal Fluid Samples

**Ioanna Tsiara [a], Mario S.P. Correia [a], Fan Yang [a], Weiming Zeng [a], Pauline Seeburger [a], Belén Hervás Povo [a], Iben Lundgaard [b,c], Manuel Menéndez-González [d,e], Miroslav Vujasinovic [f,g], J.-Matthias Löhr [g,h], and Daniel Globisch [a,*]**

[a] Department of Chemistry - BMC, Science for Life Laboratory, Uppsala University, Box 576, SE-75124, Uppsala, Sweden

[*] Corresponding author: Daniel.globisch@kemi.uu.se

[b] Department of Experimental Medical Science, Lund University, 22362 Lund, Sweden

[c] Wallenberg Centre for Molecular Medicine, Lund University, 22362 Lund, Sweden

[d] Universidad de Oviedo, Department of Medicine, Oviedo, Asturias, 33006, Spain

[e] Instituto de Investigación Sanitaria del Principado de Asturias, Oviedo, 33011, Spain

[f] Department of Medicine Huddinge, Karolinska Institute, Stockholm, Sweden

[g] Department for Upper Abdominal Diseases, Karolinska University Hospital, Stockholm, Sweden

[h] Department of Clinical Science, Intervention and Technology (CLINTEC), Karolinska Institute, Stockholm, Sweden

**Determination of glucuronidase activity**

Glucuronidase activity was tested according to the protocol described by Sigma Aldrich (S9626). To calculate the activity of glucuronidase in solution, 65 µL of H2O were mixed with 50 µL of 75 mM potassium phosphate buffer with 1% (w/v) bovine serum albumin, pH 6.8, 25 µL of 3 mM of phenolphthalein-glucuronide and 10 µL of enzyme test solution. A negative control was also tested, in which no enzyme was added. To stop the reaction, 500 µL of 200 mM glycine buffer, pH 10.4 were added. The resulting solution was transferred to a 96-well plate and the absorbance at 540 nM was measured to monitor the production of phenolphthalein. At the same time, a phenolphthalein standard curve was prepared, with a ranging quantity of 1-5 µg. The amount of phenolphthalein was plotted against the A540 value and test results were based on the measured absorbance.

The units in solution were calculated using the following equation:

$$Units/mL = \frac{(\text{µg of phenolphthalein released}) \times \text{df}}{\text{V}_\text{E}\ x\ t}$$

Details:

t – Time factor correction (Unit definition for 1 hour)

df – Protein dilution factor

$V_E$ – Volume (in mL) of purified glucuronidase used

**Figures**

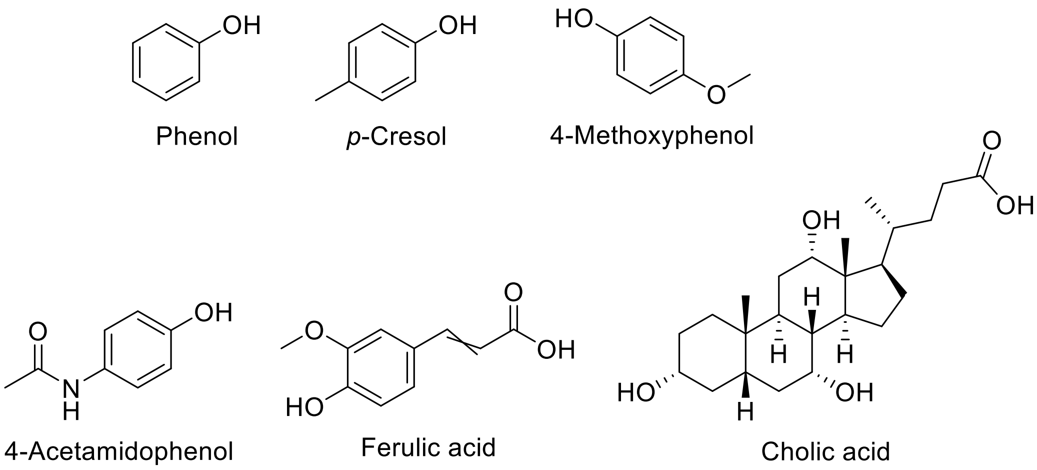


**Figure S1** Selected compounds used for the enzymatic synthesis of their corresponding glucuronides, utilizing three UGT enzymes (UGT1A1, UGT2B7 and UGT2B15).

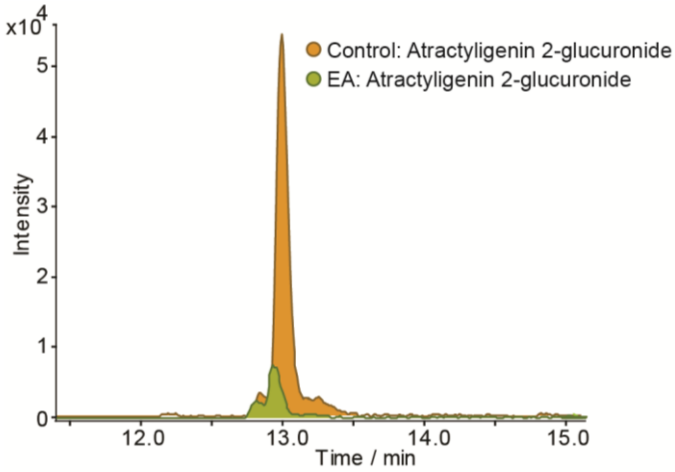


**Figure S2** Extracted Ion Chromatogram (EIC) of atractyligenin 2-glucuronide (*m/z* 497.2390) in the control group and in the enzymatic treatment group in $ESI^+$.

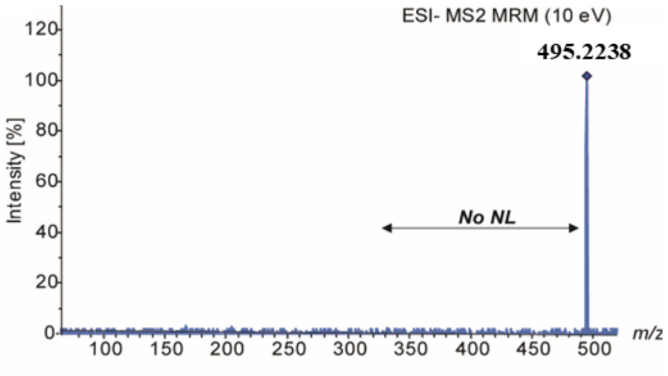


**Figure S3** MS/MS fragmentation of atractyligenin 2-glucuronide (*m/z* 495.2238) in MRM mode using 10 eV in ESI$^-$.

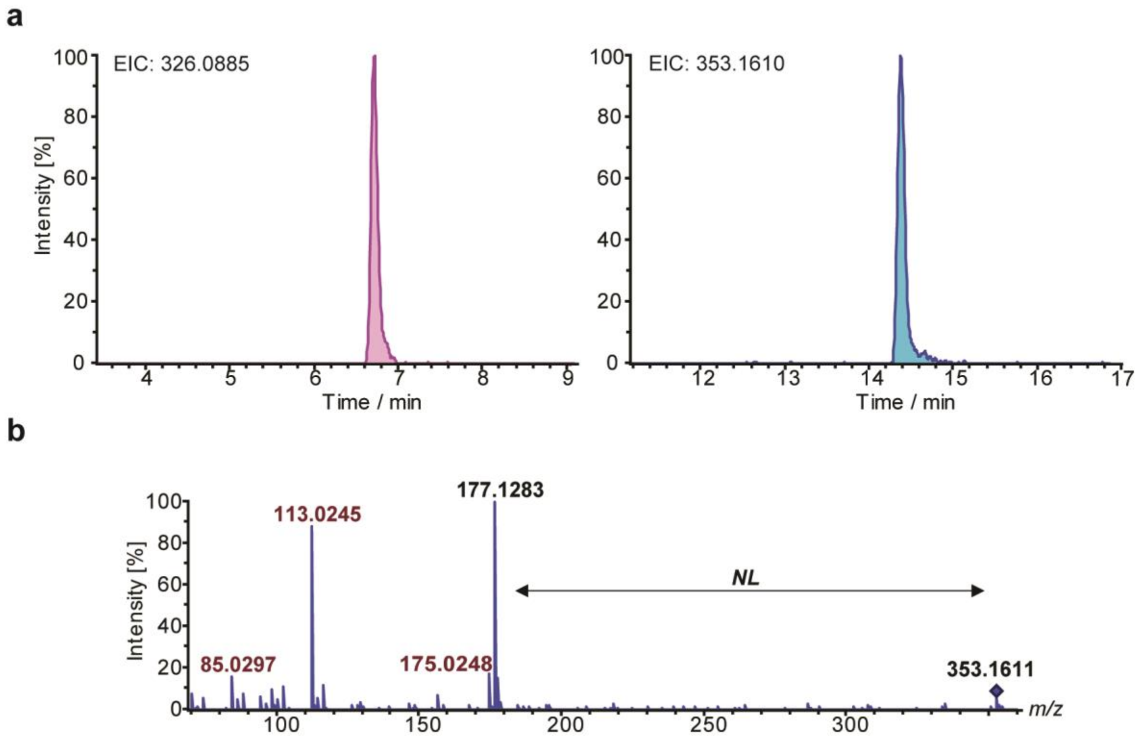


**Figure S4 a** EICs of acetaminophen glucuronide (*m/z* 326.0885) and propofol glucuronide (*m/z* 353.1610); **b** MS/MS fragmentation of propofol glucuronide validated in the CSF samples in ESI$^-$.

## Tables

**Table S1**. The six compounds tested for enzymatic synthesis of the corresponding glucuronide utilizing three UGT enzymes. The compounds were at a concentration of 250 µM.

| Compound Name | Monoisotopic Mass | Glucuronidation | | |
|---|---|---|---|---|
| | | UGT1A1 | UGT2B7 | UBT2B15 |
| Phenol | 94.0419 | - | **Yes** | **Low** |
| *p*-Cresol | 108.0575 | **Low** | **Yes** | **Yes** |
| 4-Methoxyphenol | 124.0524 | - | **Yes** | **Low** |
| 4-Acetamidophenol | 151.0633 | - | - | - |
| Ferulic acid | 194.0579 | **Yes** | - | - |
| Cholic acid | 408.2876 | **Low** | - | - |

**Table S2.** All validated glucuronidated metabolites with annotated confidence levels in the respective mode.

(Level 1: Validation with authentic synthetic or commercial standards; Level 2: Metabolite structure validation based on unambiguous matching of MS2 spectra with experimental spectra from literature or library sources; or identification of the molecular formula and MS2 fragmentation pattern comparison using computational tools; Level 3: MS2-validation of glucuronic acid moiety in the metabolite).

*Gluc: glucuronide, rt: retention time, Ctr: control group, EA: enzymatic treated group, CL: confidence level, F.P.: fingerprint fragment, NL: neutral loss, * asterisk represents a calculated molecular formula from the annotated aglycon (aglycon +* $C_6H_8O_6$*)*

| # | Monoisotopic Mass | Chemical Formula | *m/z* Gluc | rt Gluc (min) | m/z Aglycon | ID | Fold Gluc (Ctr/EA) | CL | Ionization mode | F.P. | NL | Biospecimen Location |
|---|---|---|---|---|---|---|---|---|---|---|---|---|
| 1 | 281.1111 | *$C_{10}H_{19}NO_8$ | 282.1185 | 2.06 | 106.0864 | HMDB0244975/ HMDB0253919 | 1.5 | 3 | positive | - | no | Plasma |
| 2 | 284.0894 | $C_{13}H_{16}O_7$ | 283.0820 | 10.34 | 107.0499 | HMDB0011686 | *0.9* | 1 | negative | - | yes (low) | Feces |
| 3 | 284.0900 | $C_{13}H_{16}O_7$ | 283.0826 | 10.43 | 107.0505 | HMDB0011686 | 31 | 1 | negative | 85 & 113 | yes | Plasma |
| 4 | 284.1259 | * $C_{14}H_{20}O_6$ | 285.1333 | 13.19 | 109.1012 | HMDB0246592 | 2.3 | 3 | positive | - | yes (low) | Feces |
| 5 | 290.0753 | *$C_{10}H_{14}N_2O_8$ | 289.0679 | 5.08 | 113.0358 | HMDB0060245 | 1.4 | 3 | negative | - | no | Feces |
| 6 | 295.1203 | $C_{10}H_{21}N_3O_5S$ | 294.1129 | 11.98 | 118.0808 | - | 108 | 3 | negative | 85 & 113 | no | Plasma |
| 7 | 304.6762 | $C_{32}H_{51}NO_{10}$ | 303.6688 | 16.13 | 127.6367 | CID171038641 | 74 | 3 | negative | 85 & 113 | no | Plasma |
| 8 | 305.1606 | $C_{12}H_{23}N_3O_6$ | 304.1532 | 15.68 | 128.1211 | - | 91 | 3 | negative | 85 & 113 | no | Plasma |
| 9 | 308.0899 | $C_{15}H_{16}O_7$ | 307.0825 | 10.59 | 131.0504 | HMDB0003441/ HMDB0032072 | 1.4 | 3 | negative | 85 (low) & 113 | no | Feces |
| 10 | 310.8840 | - | 309.8766 | 16.30 | 133.8445 | - | 1.9 | 3 | negative | 85 & 113 (low) | no | Plasma |
| 11 | 312.6736 | $C_{32}H_{51}NO_{11}$ | 311.6662 | 15.68 | 135.6341 | HMDB0002579 | 49.9 | 3 | negative | 85 (low) & 113 | no | Plasma |
| 12 | 327.0955 | $C_{14}H_{17}NO_8$ | 328.1029 | 7.06 | 152.0708 | HMDB0010316 | 34.2 | 2 | positive | - | yes | Plasma |
| | | | 326.0881 | 6.78 | 150.0560 | | 1732 | 2 | negative | 85 & 113 | yes | |
| 13 | 327.0959 | $C_{14}H_{17}NO_8$ | 326.0885 | 6.72 | 150.0564 | HMDB0010316 | 423 | 2 | negative | 85 & 113 & 175 | yes | CSF |
| 14 | 332.1838 | $C_{16}H_{28}O_7$ | 331.1764 | 14.89 | 155.1443 | HMDB0254445/ CID117762847 | 20 | 3 | negative | 85 & 113 | no | Plasma |
| 15 | 343.1269 | $C_{15}H_{21}NO_8$ | 344.1343 | 6.30 | 168.1022 | CID45040235 | 1.5 | 3 | positive | - | no | Plasma |
| | | | 342.1195 | 5.59 | 166.0874 | | 2.7 | 3 | negative | - | no | |
| 16 | 343.1295 | $C_{15}H_{21}NO_8$ | 344.1369 | 6.05 | 168.1048 | CID45040235 | 1.5 | 3 | positive | - | no | Feces |
| 17 | 354.1684 | $C_{18}H_{26}O_7$ | 353.1610 | 14.39 | 177.1289 | HMDB0060933 | *0.9* | 2 | negative | 85 & 113 & 175 | yes | CSF |

| | | | | | | | | | | | | |
|---|---|---|---|---|---|---|---|---|---|---|---|---|
| 18 | 380.1116 | *$C_{18}H_{20}O_9$ | 379.1042 | 9.23 | 203.0721 | HMDB0033303/ HMDB0035095 | 1.4 | 3 | negative | 85 (low) & 113 | no | Feces |
| 19 | 384.0880 | *$C_{17}H_{20}O_8S$ | 383.0806 | 10.60 | 207.0485 | HMDB0034759 | 1.9 | 3 | negative | - | no | Feces |
| 20 | 384.0915 | *$C_{14}H_{24}O_8S_2$ | 383.0841 | 11.13 | 207.0520 | HMDB0257963/ HMDB0012210/ HMDB0060589 | 1.8 | 3 | negative | - | no | Feces |
| 21 | 416.2052 | $C_{20}H_{32}O_9$ | 415.1978 | 14.21 | 239.1657 | - | 8.9 | 3 | negative | 85 (low) & 113 | no | Plasma |
| 22 | 462.2254 | $C_{25}H_{34}O_8$ | 463.2328 | 16.51 | 287.2007 | CID5460852/H MDB0010337 | 8.8 | 3 | positive | - | yes | Plasma |
| 23 | 463.1846 | $C_{23}H_{29}NO_9$ | 464.1920 | 13.28 | 288.1599 | HMDB0060821/ CID129627161/ CID9825634/CI D46780422/CID 46780423/CID4 5358980/CID71 750840/CID117 065138/CID998 1841/CID10136 0603 | 31 | 3 | positive | - | yes | Plasma |
| 24 | 463.1849 | $C_{23}H_{29}NO_9$ | 464.1923 | 13.07 | 288.1602 | HMDB0060821/ CID129627161/ CID9825634/CI D46780422/CID 46780423/CID4 5358980/CID71 750840/CID117 065138/CID998 1841/CID10136 0603 | 59 | 3 | positive | - | yes | Plasma |
| 25 | 472.2241 | $C_{25}H_{32}N_2O_7$ | 473.2315 | 10.83 | 297.1994 | CID46781815/H MDB0060717 | 1.2 | 3 | positive | - | no | Feces |
| 26 | 483.0951 | $C_{15}H_{21}N_3O_{15}$ | 482.0877 | 8.67 | 306.0556 | - | 2171 | 3 | negative | 85 & 113 | yes (low) | Plasma |
| 27 | 496.2315 | $C_{25}H_{36}O_{10}$ | 495.2241 | 12.76 | 319.1920 | HMDB0240481/ CID156960878 | 1025 | 2 | negative | 85 (low) & 113 (low) | no | Plasma |
| | | | 497.2390 | 13.01 | 321.2069 | | 13 | 2 | positive | | yes | |
| 28 | 508.2308 | $C_{26}H_{36}O_{10}$ | 507.2234 | 16.15 | 331.1913 | - | 10 | 3 | negative | 85 (low) & 113 (low) | yes | Plasma |
| 29 | 540.2573 | $C_{27}H_{40}O_{11}$ | 539.2499 | 13.63 | 363.2178 | HMDB0010357 | 18 | 3 | negative | 85 (low) & 113 | no | Plasma |

| | | | | | | | | | | | | |
|---|---|---|---|---|---|---|---|---|---|---|---|---|
| 30 | 542.2730 | $C_{27}H_{42}O_{11}$ | 541.2656 | 13.41 | 365.2335 | HMDB0010320 | 536 | 3 | negative | 85 & 113 | no | Plasma |
| 31 | 568.1978 | $C_{23}H_{36}O_{16}$ | 567.1904 | 14.00 | 391.1583 | - | 816 | 3 | negative | 85 & 113 | no | Plasma |
| 32 | 608.3218 | *$C_{32}H_{48}O_{11}$ | 609.3292 | 14.30 | 433.2971 | HMDB0014792/ HMDB0244526 | 1.5 | 3 | positive | - | no | Feces |

**Table S3.** Annotations and chemical classes of the identified glucuronidated metabolites.

** Asterisk represents a calculated molecular formula from the annotated aglycon (aglycon +* $C_6H_8O_6$*)*

| Monoisotopic Mass | Chemical Formula | Name | Class |
|---|---|---|---|
| 281.1111 | *$C_{10}H_{19}NO_8$ | 2-Amino-2-methyl-1,3-propanediol / L-Threoninol | Amino alcohols |
| 284.0894 | $C_{13}H_{16}O_7$ | *p*-Cresol glucuronide | Phenols |
| 284.1259 | *$C_{14}H_{20}O_6$ | 4-Vinylcyclohexene | Cycloalkenes |
| 290.0753 | *$C_{10}H_{14}N_2O_8$ | L-3-Cyanoalanine | Amino acids, peptides, and analogues |
| 295.1203 | $C_{10}H_{21}N_3O_5S$ | - | Unknown |
| 304.6762 | $C_{32}H_{51}NO_{10}$ | Glycolithocholic acid glucuronide | Steroids and derivatives |
| 305.1606 | $C_{12}H_{23}N_3O_6$ | - | Unknown |
| 308.0899 | *$C_{15}H_{16}O_7$ | Cinnamaldehyde | Benzaldehydes |
| 312.6736 | $C_{32}H_{51}NO_{11}$ | Glycochenodeoxycholic acid glucuronide | Steroids and derivatives |
| 327.0955 | $C_{14}H_{17}NO_8$ | Acetaminophen glucuronide | Phenols |
| 332.1838 | $C_{16}H_{28}O_7$ | Menthol glucuronide / Citronellyl glucuronide | Terpenoids |
| 343.1269 | $C_{15}H_{21}NO_8$ | Phenylephrine glucuronide | Phenols |
| 354.1684 | $C_{18}H_{26}O_7$ | Propofol glucuronide | Phenols |
| 380.1116 | *$C_{18}H_{20}O_9$ | Anofinic acid / 7-Ethoxy-4-methyl-2H-1-benzopyran-2-one | Coumarins and derivatives |
| 384.088 | *$C_{17}H_{20}O_8S$ | Methyl 5-(1-Propynyl)-2-thiophenepropanoate | Thiophenes and derivatives |
| 384.0915 | *$C_{14}H_{24}O_8S_2$ | - | Unknown |
| 416.2052 | $C_{20}H_{32}O_9$ | - | Unknown |
| 462.2254 | $C_{25}H_{34}O_8$ | 6-Dehydrotestosterone glucuronide | Steroids and derivatives |
| 463.1846 | $C_{23}H_{29}NO_9$ | Dihydromorphine-3-glucuronide | Alkaloids and derivatives |
| 472.2241 | $C_{25}H_{32}N_2O_7$ | 2-Hydroxy-imipramine glucuronide | Alkaloids and derivatives |
| 483.0951 | $C_{15}H_{21}N_3O_{15}$ | - | Unknown |
| 496.2315 | $C_{25}H_{36}O_{10}$ | Atractyligenin 2-glucuronide | Terpenoids |
| 508.2308 | $C_{26}H_{36}O_{10}$ | - | Unknown |
| 540.2573 | $C_{27}H_{40}O_{11}$ | Tetrahydroaldosterone-3-glucuronide | Steroids and derivatives |
| 542.273 | $C_{27}H_{42}O_{11}$ | Cortolone-3-glucuronide | Steroids and derivatives |
| 568.1978 | $C_{23}H_{36}O_{16}$ | - | Unknown |
| 608.3218 | *$C_{32}H_{48}O_{11}$ | Latanoprost / isopropyl 7-[3,5-dihydroxy-2-(3-hydroxy-5-phenylpentyl)cyclopentyl]hept-5-enoate | Eicosanoids |
